# Sugammadex and Reversal of Neuromuscular Block in Adult Patient with Duchenne Muscular Dystrophy

**DOI:** 10.1155/2014/680568

**Published:** 2014-03-13

**Authors:** Ahmed Abdelgawwad Wefki Abdelgawwad Shousha, Maria Sanfilippo, Antonio Sabba, Paolo Pinchera

**Affiliations:** Department of Anesthesiology and Intensive Care, Sapienza University, Viale del policlinico 155, 00161 Rome, Italy

## Abstract

Duchenne's muscular dystrophy (DMD) is the most common and severe form of myopathy. Patients with DMD are more sensitive to sedative, anesthetic, and neuromuscular blocking agents which may result in intraoperative and early postoperative cardiovascular and respiratory complications, as well as prolonged recovery from anesthesia. In this case report, we describe a 25-year-old male patient admitted for cholecystectomy under general anesthesia. We induced our anesthesia by oxygen, propofol, fentanyl, and rocuronium bromide. Maintenance was done by fentanyl, rocuronium bromide, sevoflurane, and O_2_. We report in this case the safety use of sugammadex to antagonize the neuromuscular block and rapid recovery in such category of patients.

## 1. Introduction 

Duchenne muscular dystrophy (DMD) is a rare genetic X-linked recessive disorder but it is one of the most frequent genetic conditions affecting approximately 1 in 3,500 male births worldwide. It is usually recognized between three and six years of age. DMD is characterized by weakness and wasting (atrophy) of the muscles of the pelvic area followed by the involvement of the shoulder muscles. As the disease progresses, muscle weakness and atrophy spread to affect the trunk and forearms and gradually progress to involve additional muscles of the body [1, 2].

The anesthetic management of these patients is complicated not only by muscle weakness but also by cardiac and pulmonary manifestations.

However there is no definite recommendation for either general or regional anaesthesia.

Succinylcholine and volatile anaesthetics have been best avoided because there is a risk of hyperkalemic cardiac arrest or severe rhabdomyolysis [3].

Some authors have suggested intubation and anesthesia without resorting to muscle relaxants, in order to avoid postoperative respiratory failure related to the usage of muscle relaxants and the other complications induced by acetylcholinesterase inhibitors. However, anesthesia without muscle relaxants might not always be suitable for some surgical procedures like such as in our patient [4].

Case reports in patients with myasthenia gravis document the successful use of sugammadex (six case reports). For other rare muscular diseases like Duchenne muscular dystrophy recent reports document the successful reversal of rocuronium with sugammadex in pediatric patients [5–9].

And in this case report we document the sugammadex safety in an adult Duchenne disease patient.

## 2. Case Presentation

A 25-year-old male with DMD with a modified Barthel index of 23 (Barthel index is an ordinal scale used to measure performance in activities of daily living) [10] (BMI 25,6,ASA III) was scheduled for open cholecystectomy under general anesthesia. The surgery duration was about 240 minutes and this prolongation was due to further undiagnosed stenosis of the biliary tract. His medical history revealed DMD disability, moderate restrictive pulmonary dysfunction, mild hypokalemia, and hypertension.

His preoperative laboratory tests were hemoglobin 13.9 g^−1^, hematocrit 43.5%, platelets 202,000 mm^−3^, sodium 141 mmom·L^−1^, potassium 3 mmol·L^−1^, magnesium 0.58 mg·dL^−1^, creatinine 0.06 mg·dL^−1^, total calcium 8.72 mg·dL^−1^, lactic dehydrogenase (LDH) 230 U·L^−1^, direct bilirubin 230 U·L^−1^, and alkaline phosphatase 130 U·L^−1^.

For the common difficulty to obtain a peripheral venous access in such patients, a central venous access was established by ultrasound guided cannulation of the internal right jugular vein.

In the preoperative room we prepared our patient by antibiotics prophylaxis: ciprofloxacin 2 gm; metronidazole 500 mg; and an antiemetic agent ondansetron 4 mg.

Our patient was monitored by pulse oximetry, expiratory capnography, invasive and noninvasive blood pressure, electrocardiogram, neuromuscular transmission by train-of-four repeated every 12 seconds at the adductor pollicis muscle (TOF Guard Organon Teknika B.V, Boxtel, The Netherlands), and diuresis.

We induced our anesthesia by oxygen, propofol 150 mg, fentanyl 200 mcg, and rocuronium bromide 10 mg, and then we proceeded to a rapid sequence endotracheal intubation (tube diameter was 7.5 mm).

The maintenance of the anesthesia was achieved by fentanyl in a total dose of 400 mcg (200-100-100), rocuronium bromide 5 mg repeated every 45 minutes at T4/T1 recovery of 25%, sevoflurane 2%, and O_2_ 40% in air. The fluid replacement was calculated depending on his diuresis, plasma fluid, and intraoperative blood loss and he had received a total fluids amount of Ringer Lactate 1500 mL and Nacl 0.9% 1000 mL. He was mechanically ventilated with these parameters: IPPV with respiratory frequency 12 incursions per minute, tidal volume of 550 mL, PEEP 5 cm H_2_O, and inspiratory/expiratory time ratio 1 : 2.

Blood gas analysis was performed twice (at the middle of the surgery and one hour after the end of surgery) by obtaining blood samples through the arterial catheter used for invasive blood pressure monitoring. For hypokalemia we administered KCl 40 mEq (Tables 1 and 2).

At the end of surgery and immediately before the emergence phase we registered that TOF ratio was 25%; we administrated Sugammadex at a single dose of 150 mg; in 5 minutes we obtained a TOF ratio of 75% increasing; for another 5 minutes TOF ratio reached 90%; the patient was extubated with careful monitoring for his cardiovascular and respiratory functions.

After 15 minutes we got the complete recovery of our patient; he was awake with excellent and stabile both hemodynamic and respiratory functions.

## 3. Conclusion 

However there are few studies which can confirm the safety use of Sugammadex in patients with muscular dystrophic diseases and however mainly these few studies were implicated on pediatric population and especially in patients with myasthenia gravis. In our case we had the fortune to confirm the effectiveness and the safety of the drug in an adult patient with Duchenne dystrophy as we registered the rapid emergence without any postoperative residual curarization.

Also we recommend whenever it is necessary the use of rocuronium in such category of patient; the Sugammadex can provide a rapid and safe reversal moderate neuromuscular block after administration of rocuronium in dose of 2 mg·kg^-1.^

## Figures and Tables

**Table 1:** Hemogasanalysis at the middle of the surgery.

Temperature (37.0°C)	
pH	7.37
pCO_2_	34 mmHg
pO_2_	322 mmHg
Na^+^	141 mmol/L
K^+^′	2.8 mmol/L
Ca^++ ^	1.48 mmol/L
Glu	95 mg/dL
Lac	0.5 mmol/L

Oximeter	
tHb	12.4 g/dL
°2 Hb	96.3%
COHb	1.8%
MetHb	1.6%
HHb	0.3%
5°2	99.7%

Derivatives	
TCO_2_	20.7 mmol/L
BEecf	−5.6 mmol/L
BE (B)	−4.8 mmol/L
Ca^++^ (7.4)	1.46 mmol/L
SO_2_ (C)	99.9%
HCO_3_-(c)	19.7 mmol/L
HCO_3__standard_	21.2 mmol/L
Hct (c)	37%

**Table 2:** Hemogasanalysis 1 hour after the end of surgery.

Temperature (37.0°C)	
pH	7.24
pCO_2_	45 mmHg
pO_2_	137 mmHg
Na^+^	142 mmol/L
K^+^′	4.9 mmol/L
Ca^++^	1.39 mmol/L
Glu	109 mg/dL
Lac	0.7 mmol/L

Oximeter	
tHb	13.9 g/dL
°2 Hb	96.4%
COHb	2.1%
MetHb	1.2%
HHb	0.3%
5°2	99.7%

Derivatives	
TCO_2_	20.7 mmol/L
BEecf	−8.1 mmol/L
BE (B)	−8.0 mmol/L
Ca^++^ (7.4)	1.30 mmol/L
SO_2_ (C)	98.6%
HCO_3_-(c)	19.3 mmol/L
HCO_3__standard_	19.3 mmol/L
Hct (c)	42%
